# Evaluating the Effectiveness of School Closure in COVID-19–Related Syndromes From Community-Based Syndromic Surveillance: Longitudinal Observational Study

**DOI:** 10.2196/44606

**Published:** 2023-12-15

**Authors:** Ping-Chen Chung, Kevin J Chen, Hui-Mei Chang, Ta-Chien Chan

**Affiliations:** 1 Department of Dentistry Puzi Hospital Ministry of Health and Welfare Chiayi County Taiwan; 2 Department of Health Taipei City Government Taipei City Taiwan; 3 Research Center for Humanities and Social Sciences Academia Sinica Taipei City Taiwan; 4 Institute of Public Health School of Medicine National Yang Ming Chiao Tung University Taipei City Taiwan

**Keywords:** school closure, COVID-19, syndromic surveillance, outpatient, mobility

## Abstract

**Background:**

During the COVID-19 pandemic, a school closure policy was adopted to prevent cluster transmission in schools and subsequent household transmission. However, the effectiveness of school closure is not consistent in studies conducted in different countries.

**Objective:**

This study aimed to explore the association between school closure and the daily standardized incidence of COVID-19–related syndromes in an outpatient syndromic surveillance system.

**Methods:**

We calculated the incidence of COVID-19–related syndromes derived from a community-based syndromic surveillance system between the first week of January and the second or fourth weeks after school closure in 2021 and 2022 in Taipei City, Taiwan. The effect of school closure on the standardized incidence of COVID-19–related syndromes was evaluated by interrupted time series analysis using an autoregressive integrated moving average with a distributed lag function. The exogenous variables were changes in human mobility measured by Google COVID-19 community mobility reports. Furthermore, the models quantified the influence of different age groups and the hierarchy of medical facilities, such as clinics or community hospitals.

**Results:**

School closure was only negatively and significantly associated with the overall standardized incidence of COVID-19–related syndromes in 2021 for 2 weeks after the intervention (coefficient −1.24, 95% CI −2.40 to −0.08). However, in different age groups, school closure had a significantly negative association with the standardized incidence among people aged 13-18 years and ≥65 years for 2 weeks after the intervention in clinics in 2021. In community hospitals, school closure was significantly positively associated with the standardized incidence among people aged 19-24 years in 2021. In 2022, 2 weeks after the intervention, school closure had a significantly negative association with the standardized incidence among people aged 0-6, 7-12, and 19-24 years in community hospitals and aged >45 years in clinics. Furthermore, the standardized incidence was positively associated with movement change toward grocery and pharmacy stores in all age groups in 2022. In addition, movement changes toward residences were significantly positively associated with the standardized incidence among all age groups.

**Conclusions:**

Overall, school closure effectively suppresses COVID-19–related syndromes in students owing to the reduction of physical contact. In addition, school closure has a spillover effect on elderly people who stay at home.

## Introduction

Before the massive COVID-19 vaccination campaign for school children, school closure was the most adopted nonpharmaceutical policy to prevent cluster transmission in schools and subsequently household transmission. However, the effectiveness of school closure is not consistent among studies conducted in different countries [1]. With the adaptation of SARS-CoV-2 to humans, the highly attacked population has shifted from elderly people and adults to school children [2]. As epidemiological characteristics change, the evaluation of the school closure policy may be different. One study conducted during the early phase of the pandemic in 2020 [3] examined the effect of policies, including school closure, on reducing local transmission of COVID-19. At that time, the majority of the infected population was adults. Therefore, the effectiveness of school closure was not significant. However, as the pandemic progressed, the low vaccination coverage of school children and the reopening of schools [4] caused the infection rate among school children to surge. In the United States, children aged <18 years accounted for 17.3% of reported cases of COVID-19 until August 30, 2022 [5]. In Taiwan, up to calendar week 31 in 2022, the proportion of confirmed cases of COVID-19 among those aged under 20 years was 13.9% [6]. The attack rate and susceptibility to COVID-19 infection are lower in children than in adults according to current evidence [7]. Current research suggests that COVID-19 angiotensin-converting enzyme 2 receptors are scarce in the respiratory tract of children, leading to fewer receptors for viruses to bind to [8]. Although there appear to be few confirmed cases among children, some data suggest that children are more likely to be asymptomatic or mild and that they are not initially tested, which leads to underreported cases [9]. Children may still be a source of infection transmission [9].

The prevention of infection and severe complications, especially in children, remains a public health issue. Nonpharmaceutical interventions (NPIs) are effective strategies to mitigate the spread of epidemics in the community. Strategies include maintaining personal hygiene, wearing masks, social distancing, daily temperature measurements, and symptom monitoring. School closure or class suspension as an NPI is often considered an option for suppressing the spread of influenza and enterovirus epidemics [10-12]. School closure can reduce transmission first among children and then in the community [13]. A published systematic review noted that the mean reduction in the peak of the influenza epidemic was 29.65% (SD 23.63) after implementing school closure [10]. The earlier implementation of school closure is more likely to reduce and delay the peak of the influenza epidemic [10]. School closure can decrease the number of daily contacts between schoolmates in close proximity. A simulation study showed that reproduction number and age-specific susceptibility to infection influenced the policy effect of school closure [14]. School closure is less effective under the condition of a larger reproduction number (>1.8) and a population with less susceptibility to infection [14]. Greater transmissibility (reproduction number) and lower susceptibility to COVID-19 in children are opposed to influenza. The effects of school closure to combat COVID-19 at different times and in different countries are still disputed [1].

A recent systematic review revealed that the impact of school closure on COVID-19 is smaller than that of other social distancing interventions, and school closure alone only prevents 2%-4% of deaths [1]. In the fall of 2020 in Croatia, an association was found between school closure and COVID-19 morbidity and mortality, while in the winter of 2021, the association was insignificant [15]. According to a United Nations International Children’s Emergency Fund (UNICEF) report, from March 2020 to February 2021, schools for more than 168 million children worldwide were closed for nearly a year [16]. During the COVID-19 pandemic, school closure policies have been widely implemented worldwide to reduce exposure rates and transmission risks ahead of mass vaccination. According to a systematic review of school closure during the pandemic, school closure and in-school mitigation measures were associated with reduced COVID-19 transmission in the community [17]. However, they also noted that assessing the impact of school closure is challenging because many nonpharmacological interventions are being implemented simultaneously, and the potential negative impacts on students’ mental and physical health were also widely discussed [17,18]. School closure can negatively influence children’s physical and mental health, and education, and can have an economic impact on working parents. Therefore, before deciding on the implementation of school closure, we must consider its effects and costs. The timing of the intervention to mitigate emerging infectious diseases was referenced by prompt surveillance. Sentinel-based surveillance for monitoring disease activity has been widely used in different countries. Sentinel surveillance can detect early aberrations in the daily incidence of a disease or the outpatient consultation rate [19]. In this study, we built community-based syndromic surveillance (ie, Sentinel Plus) in sentinel clinics and community hospitals since June 2018 in Taipei City, Taiwan. The advantages of Sentinel Plus can be used to monitor a variety of syndromes and detect changes across 7 age groups and health care facility levels, including community hospitals and clinics [19]. Sentinel Plus is designed for daily automated symptom monitoring of 34 current symptom groups in an outpatient setting to complement emergency room surveillance. In our previous study [19], Sentinel Plus performed better than other surveillance systems in early aberration detection in influenza-like illnesses and enterovirus-like syndromes. Owing to the lack of empirical evidence on the influence of the COVID-19 epidemic during the implementation of school closure, this study aimed to explore the association between school closure and the standardized incidence of COVID-19–related syndromes derived from daily outpatient syndromic surveillance data. Furthermore, we elucidated the association between 7 different age stratifications and the hierarchy of medical facilities. Moreover, the effects of school closure were explored in different extents of mobility changes between 2021 and 2022.

## Methods

### Data Source

We collected specific syndromic groups daily from a community-based enhanced sentinel surveillance system named “Sentinel Plus,” which has been designed for the early detection of aberrations of epidemics in clinics and community hospitals since June 2018 [19]. After November 2020, Sentinel Plus monitors expanded the syndromic groups from 23 to 34 syndromes owing to the COVID-19 pandemic. The specific International Classification of Diseases, 10th Revision (ICD-10) diagnoses from the hospital information systems of participating clinics and community hospitals were computed on-site and aggregated into 34 syndromic groups and 7 age groups without any patient identifiers. The aggregated data were then sent to Sentinel Plus through a secure channel.

In this study, we used COVID-19–related syndromes from Sentinel Plus. ICD-10 diagnoses of daily visits from December 2020 to June 2022 were obtained from 130 participating clinics, 12 health centers, and 8 community hospitals in Taipei City. The definition of COVID-19–related syndromes was discussed with the family physician and infectious disease physician, and was therefore identified by ICD-10 codes, including R05, R06.02, R50.9, R43.0, R43.1, R43.2, R43.8, R43.9, R19.7, J06.9, J12.89, J34.89, R07.0, R51, and R19.7. Although the confirmed diagnosis of COVID-19 was implemented in the system in November 2020, reimbursement for the diagnosis of COVID-19 through the routine national health insurance system began at the end of May 2021. From June 2021 to late May 2022, COVID-19 testing by polymerase chain reaction (PCR) was only implemented in designated hospitals and community screening stations. Clinics did not make a diagnosis of COVID-19 but transferred possible patients to hospitals and screening stations during that time. Therefore, we did not use a COVID-19–confirmed diagnosis as the primary outcome.

In addition to the surveillance data, we also incorporated the policy of school closure in Taipei City and the Google COVID-19 Community Mobility Report for analysis. School closure information was obtained from a press release from the Department of Education of Taipei City Government. Daily percentage changes in people’s visits to and staying time in the 6 categories of places were obtained from the COVID-19 Community Mobility Report [20]. The data charted movement trends over time compared to baseline days, which was the median value for the corresponding day of the week during the 5-week period from January 3 to February 6, 2020. Categories for grouping places with similar characteristics for social distancing guidance included grocery stores and pharmacies, parks, transit stations, retail and recreation, residences, and workplaces. Daily alert levels for COVID-19 from the Central Epidemic Command Center were downloaded from the Taiwan Centers for Disease Control (CDC) [21] as a reference. In Sentinel Plus, the age group was classified into 7 categories as follows: 0-6, 7-12, 13-18, 19-24, 25-44, 45-64, and ≥65 years.

### Ethics Approval

The study was approved by the Institutional Review Board of the Biomedical Science Research, Academia Sinica (AS-IRB-BM-18017).

### Data Analysis

In Taipei City, school closure and distance learning at home were implemented from May 18, 2021, to July 2, 2021. The time intervals from January 1, 2021, to June 1, 2021 (T1-1) and June 15, 2021 (T1-2) were analyzed. In 2022, Taipei’s school closure was from May 23 to June 5. The time intervals from January 1, 2022, to June 6, 2022 (T2-1) and June 20, 2022 (T2-2) were analyzed. We assumed that the school closure on COVID-19–related syndromes lasted 1-14 days, 2-15 days, or 3-16 days after the intervention. We explored the effect of school closure on the standardized incidence of COVID-19–related syndromes, which were derived from Sentinel Plus by autoregressive integrated moving average interrupted time series-distributed lag (ARIMAITS-DL) [22], and quantified the influence of different age groups and the hierarchy of medical facilities, such as clinics and community hospitals. Moreover, the effects of school closure under movement changes between 2021 and 2022 were explored. Incidence was defined as the daily counts for the diagnosis of COVID-19–related syndromes divided by the total daily outpatient visits multiplied by 1000. Patients may be diagnosed with multiple ICD codes in the same syndromic group, and thus, the incidence may be >1000. Owing to the low number of outpatient visits on weekends and holidays, we removed daily visits below 200 from our analysis and in our plot. The ARIMAITS-DL model with exogenous variables simultaneously models both the unclear intervention time and the distributed effect of the intervention over time. It is assumed that the intervention effect is uniformly distributed over time. We checked the collinearity between the 6 categories of grouping places from the Google Community Mobility Report and deleted categories, including retail, recreation, and transit stations, owing to a variance inflation factor >10. Parks were also excluded because parks usually refer to official national parks and not the general outdoors. Workplaces were excluded because children and retirees rarely go there. Exogenous variables were grocery stores, pharmacies, and residences. The regression parameters were estimated using the maximum likelihood method. The appropriate autoregressive integrated moving average (ARIMA) parameters (p,d,q) were selected using the Akaike information criterion (AIC) and model fitting with different age groups and hierarchies of medical facilities. We checked the sensitivity of the results by changing the duration of the school closure effect with model-fitting statistics using the AIC. Finally, the residuals of the selected model and autocorrelation were tested using a residual plot and the Ljung-Box test, respectively. If autocorrelation still existed in the residuals, different autoregressive or moving average orders were chosen. All analyses were performed using R software (version 4.2.1) [23], including the forecast [24] and ggplot2 [25] packages.

## Results

**Figure 1:**
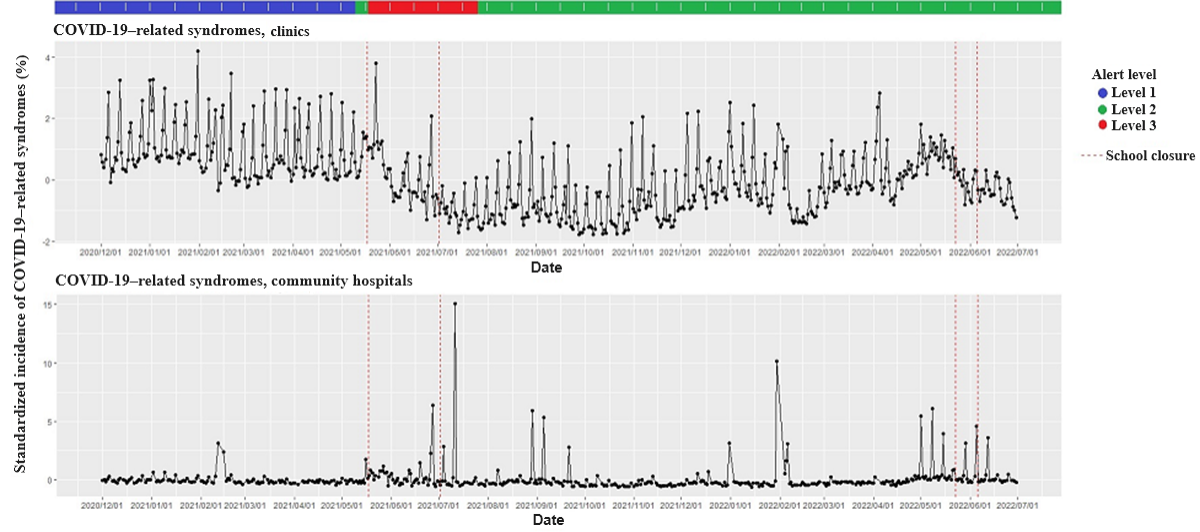
Time series plot of COVID-19–related syndromes in Taipei City.

In the first observation period of 2021 (T1-1 and T1-2), the alert level of COVID-19 was elevated to Level 3 on May 19, 2021. In the second observation period in the first half of 2022 (T2-1 and T2-2), the alert level of COVID-19 was Level 2 (Figure 1). The standardized incidence of the diagnosis of COVID-19–related syndromes separately from clinics and community hospitals in the observed period is illustrated in Figure 1. The epi-curve varied regularly on workdays and weekends, and the trend has been decreasing since school closure, particularly in clinics, in 2021.

The upper panel represents data from clinics, and the lower panel represents data from community hospitals. The color of the first row represents 3 different alert levels in Taipei City issued by the Central Epidemic Command Center (CECC) of Taiwan. Blue represents Level 1, green represents Level 2, and red represents Level 3.

The epi-curves marked between the 2 red dashed lines are the periods of school closure. Daily visits below 200 (weekends and spring festivals) were removed from the plot. Removed dates in clinics: February 12, 2021; February 01, 2022; and February 02, 2022. Removed dates in community hospitals: December 20, 2020; February 14, 2021; July 25, 2021; September 26, 2021; October 11, 2021; November 21, 2021; December 05, 2021; December 12, 2021; January 09, 2022; and February 13, 2022.

The median incidence of COVID-19–related syndromes was higher in clinics than in community hospitals in 2021 and 2022 (Tables 1 and 2, respectively). Before the implementation of school closure, the median incidence of COVID-19–related syndromes was higher in 2021 than in 2022 in both clinics and community hospitals. In different age groups, the incidence of patients aged <18 years was relatively high in clinics. In contrast, in community hospitals, the incidence of patients aged over 25 years was relatively high in 2021 and 2022.

In the study time interval of 2021 (T1-1), the overall standardized incidence of COVID-19–related syndromes was not significantly associated with the intervention of school closure in clinics (coefficient −0.84, 95% CI −1.69 to 0.01), but was negatively associated in community hospitals (coefficient −1.24, 95% CI −2.40 to −0.08) (Table 3).

In different age groups, school closure had a significant negative association with the standardized incidence among people aged 13-18 years and ≥65 years in clinics. School closure had a significant positive association with the standardized incidence among people aged 19-24 years in community hospitals. Furthermore, the standardized incidence had significant positive associations with the change in movement toward grocery and pharmacy stores among people aged 7-12 years and 25-44 years in clinics. Furthermore, changes in movement toward residences were significantly positively associated with standardized incidence rates in all age groups, in the 13-18 and >25 age groups in clinics, and in all age groups in community hospitals. However, changes in movement toward residences were significantly negatively associated with the standardized incidence among people aged 7-18 years in community hospitals.

In the study time interval of 2021 (T1-2), the overall standardized incidence of COVID-19–related syndromes in both clinics and community hospitals was not significantly associated with the school closure intervention (clinic: coefficient −0.42, 95% CI −1.34 to 0.50; community hospital: coefficient −0.45, 95% CI −1.65 to 0.76) (Table 4). In different age groups, school closure was significantly negatively associated with the standardized incidence among people aged 0-6, 13-18, and ≥65 years in clinics. However, school closure had a significant positive association with the standardized incidence among people aged 19-24 years in community hospitals.

In the first half of 2022 (T2-1), school closure had no relationship with the overall standardized incidence in both clinics and community hospitals (clinic: coefficient −0.40, 95% CI −0.90 to 0.10; community hospital: coefficient −0.29, 95% CI −0.60 to 0.03) (Table 5). In the 45-64 and ≥65 age groups in clinics, school closure had a significant negative association with the standardized incidence. In the 0-6, 7-12, and 19-24 age groups in community hospitals, school closure had a significant negative association with the standardized incidence. Among people of all ages and those aged >25 years in clinics and people of all ages and those aged >45 years in community hospitals, changes in movement toward grocery and pharmacy stores were positively associated with the standardized incidence. In clinics, among people of all ages and those aged >19 years, movement changes toward residences had a significantly positive association with the standardized incidence. In community hospitals, among people of all ages and those aged 0-6, 7-12, 19-24, and >45 years, movement changes toward residences had a significantly positive association with the standardized incidence.

In the first half of 2022 (T2-2), school closure had no relation with the overall standardized incidence in both clinics and community hospitals (clinic: coefficient −0.29, 95% CI −0.77 to 0.19; community hospital: coefficient −0.19, 95% CI −0.48 to 0.10) (Table 6). In the 45-64 and ≥65 age groups in clinics, school closure had a significant negative association with the standardized incidence.

In 2021, during a small-scale epidemic with a soft lockdown policy in effect from May to July, school closure effectively reduced the COVID-19 incidence among preschool students, junior and senior high school students, and elders. During both time intervals, there was an increase in mobility to residences, grocery stores, and pharmacies based on the Google Community Report [20].

**Table 1:** Comparison of the daily incidence of the diagnosis of COVID-19–related syndromes before and after school closure between clinics and community hospitals in 2021.

Age	Before school closure in 2021			Two weeks after school closure in 2021			Four weeks after school closure in 2021	
	Clinic, median (IQR)	Hospital, median (IQR)	Clinic, median (IQR)		Hospital, median (IQR)	Clinic, median (IQR)		Hospital, median (IQR)
Overall	59.88 (19.22)	1.78 (0.47)	64.93 (12.35)		3.46 (0.97)	54.10 (22.89)		2.53 (1.71)
0-6 years	108.67 (18.29)	0.49 (0.29)	108.36 (21.32)		0.23 (0.45)	98.40 (26.99)		0.00 (0.24)
7-12 years	126.15 (25.89)	0.34 (0.31)	127.85 (29.46)		0.00 (0.31)	127.85 (22.71)		0.00 (0.06)
13-18 years	101.23 (32.37)	0.25 (0.55)	108.70 (48.43)		0.00 (0.47)	94.59 (39.02)		0.00 (0.06)
19-24 years	85.14 (19.58)	0.59 (0.49)	99.34 (42.19)		1.61 (2.06)	77.27 (59.28)		1.01 (1.69)
25-44 years	71.04 (14.90)	2.83 (1.09)	74.96 (21.83)		7.05 (2.17)	67.29 (38.91)		6.14 (3.20)
45-64 years	42.01 (16.98)	3.54 (1.47)	50.84 (7.48)		6.55 (1.11)	48.01 (17.37)		5.86 (3.84)
≥65 years	36.90 (20.91)	4.16 (1.79)	48.07 (11.50)		6.57 (2.93)	46.62 (13.66)		5.05 (3.89)

**Table 2:** Comparison of the daily incidence of the diagnosis of COVID-19–related syndromes before and after school closure between clinics and community hospitals in 2022.

Age	Before school closure in 2022			Two weeks after school closure in 2022			Four weeks after school closure in 2022	
	Clinic, median (IQR)	Hospital, median (IQR)	Clinic, median (IQR)		Hospital, median (IQR)	Clinic, median (IQR)		Hospital, median (IQR)
Overall	46.82 (18.10)	2.24 (1.91)	46.20 (10.97)		2.10 (0.55)	43.16 (7.01)		1.99 (0.34)
0-6 years	117.46 (17.09)	0.42 (0.27)	90.98 (45.42)		0.25 (0.38)	85.54 (17.40)		0.37 (0.26)
7-12 years	117.05 (30.00)	0.22 (0.29)	78.59 (52.81)		0.66 (0.25)	83.12 (48.34)		0.42 (0.30)
13-18 years	85.41 (40.21)	0.27 (0.58)	30.56 (68.81)		0.75 (0.44)	38.22 (42.48)		0.49 (0.50)
19-24 years	51.65 (32.97)	0.39 (0.66)	50.36 (24.44)		0.42 (0.32)	48.75 (8.29)		0.42 (0.33)
25-44 years	50.68 (16.11)	3.07 (1.36)	59.42 (15.25)		4.16 (1.71)	54.02 (11.25)		4.16 (1.72)
45-64 years	31.49 (20.66)	3.17 (2.19)	49.00 (10.89)		3.75 (1.55)	39.27 (11.46)		3.92 (1.25)
≥65 years	32.10 (21.02)	3.48 (1.61)	40.48 (11.62)		4.37 (0.64)	35.34 (7.12)		4.37 (0.89)

**Table 3:** Effect of school closure on the standardized incidence of COVID-19–related syndromes in clinics and community hospitals from January 1, 2021, to June 1, 2021.

Age and variable			Clinic (estimation), coefficient (95% CI)		Hospital (estimation), coefficient (95% CI)
**Overall**					
	ARIMA^a^ (p,d,q)	ARIMA (9,0,0)		ARIMA (3,0,0)	
	School closure	−0.84 (−1.69 to 0.01)		−1.24 (−2.40 to −0.08)^b^	
	Grocery and pharmacy percent change from baseline	0.01 (−0.01 to 0.03)		−0.01 (−0.04 to 0.01)	
	Residential percent change from baseline	0.10 (0.06 to 0.14)^b^		0.22 (0.16 to 0.27)^b^	
**0-6 years**					
	ARIMA (p,d,q)	ARIMA (7,0,0)		ARIMA (7,0,1)	
	School closure	−0.01 (−1.00 to 0.97)		−0.19 (−1.06 to 0.68)	
	Grocery and pharmacy percent change from baseline	0.02 (−0.004 to 0.04)		0.01 (−0.01 to 0.03)	
	Residential percent change from baseline	0.04 (−0.004 to 0.09)		−0.04 (−0.08 to 0.001)	
**7-12 years**					
	ARIMA (p,d,q)	ARIMA (2,0,8)		ARIMA (2,0,7)	
	School closure	−0.08 (−1.30 to 1.14)		0.81 (−0.27 to 1.88)	
	Grocery and pharmacy percent change from baseline	0.03 (0.002 to 0.06)^b^		0.02 (−0.001 to 0.05)	
	Residential percent change from baseline	0.003 (−0.05 to 0.06)		−0.12 (−0.17 to −0.07)^b^	
**13-18 years**					
	ARIMA (p,d,q)	ARIMA (0,1,1)		ARIMA (0,0,0)	
	School closure	−1.58 (−2.90 to −0.26)^b^		0.56 (−0.70 to 1.83)	
	Grocery and pharmacy percent change from baseline	−0.02 (−0.05 to 0.01)		0.03 (−0.004 to 0.05)	
	Residential percent change from baseline	0.07 (0.01 to 0.12)^b^		−0.09 (−0.16 to −0.03)^b^	
**19-24 years**					
	ARIMA (p,d,q)	ARIMA (1,0,0)		ARIMA (5,0,0)	
	School closure	0.99 (−0.31 to 2.29)		2.49 (1.63 to 3.34)^b^	
	Grocery and pharmacy percent change from baseline	0.02 (−0.01 to 0.05)		0.001 (−0.02 to 0.02)	
	Residential percent change from baseline	0.03 (−0.02 to 0.08)		−0.003 (−0.05 to 0.04)	
**25-44 years**					
	ARIMA (p,d,q)	ARIMA (0,1,7)		ARIMA (0,0,1)	
	School closure	0.65 (−0.52 to 1.83)		0.14 (−0.99 to 1.27)	
	Grocery and pharmacy percent change from baseline	0.05 (0.02 to 0.08)^b^		−0.02 (−0.04 to 0.01)	
	Residential percent change from baseline	0.05 (0.001 to 0.09)^b^		0.01 (−0.04 to 0.06)	
**45-64 years**					
	ARIMA (p,d,q)	ARIMA (6,1,2)		ARIMA (1,0,3)	
	School closure	−0.17 (−1.17 to 0.82)		0.96 (−0.22 to 2.15)	
	Grocery and pharmacy percent change from baseline	0.01 (−0.01 to 0.04)		0.01 (−0.02 to 0.03)	
	Residential percent change from baseline	0.10 (0.06 to 0.14)^b^		0.06 (−0.0001 to 0.11)	
≥**65 years**					
	ARIMA (p,d,q)	ARIMA (8,0,0)		ARIMA (0,0,0)	
	School closure	−1.30 (−2.06 to −0.55)^b^		0.68 (−0.58 to 1.93)	
	Grocery and pharmacy percent change from baseline	−0.002 (−0.02 to 0.01)		−0.02 (−0.05 to 0.01)	
	Residential percent change from baseline	0.15 (0.12 to 0.19)^b^		0.06 (−0.004 to 0.12)	

^a^ARIMA: autoregressive integrated moving average.

^b^*P*<.05.

**Table 4:** Effect of school closure on the standardized incidence of COVID-19–related syndromes in clinics and community hospitals from January 1, 2021, to June 15, 2021.

Age and variable			Clinic (estimation), coefficient (95% CI)		Hospital (estimation), coefficient (95% CI)
**Overall**					
	ARIMA^a^ (p,d,q)	ARIMA (6,1,2)		ARIMA (1,0,2)	
	School closure	−0.42 (−1.34 to 0.50)		−0.45 (−1.65 to 0.76)	
	Grocery and pharmacy percent change from baseline	0.03 (0.01 to 0.06)^b^		−0.01 (−0.04 to 0.01)	
	Residential percent change from baseline	0.08 (0.05 to 0.12)^b^		0.05 (−0.02 to 0.11)	
**0-6 years**					
	ARIMA (p,d,q)	ARIMA (9,0,0)		ARIMA (7,0,1)	
	School closure	−0.90 (−1.73 to −0.06)^b^		−0.43 (−1.14 to 0.27)	
	Grocery and pharmacy percent change from baseline	0.01 (−0.01 to 0.03)		0.01 (−0.01 to 0.03)	
	Residential percent change from baseline	0.03 (−0.01 to 0.07)		−0.04 (−0.07 to 0.00)	
**7-12 years**					
	ARIMA (p,d,q)	ARIMA (2,0,8)		ARIMA (0,0,6)	
	School closure	−0.05 (−1.00 to 0.90)		0.61 (−0.30 to 1.52)	
	Grocery and pharmacy percent change from baseline	0.02 (−0.01 to 0.05)		0.01 (−0.02 to 0.04)	
	Residential percent change from baseline	0.01 (−0.03 to 0.06)		−0.10 (−0.14 to −0.05)^b^	
**13-18 years**					
	ARIMA (p,d,q)	ARIMA (2,0,6)		ARIMA (0,0,0)	
	School closure	−1.43 (−2.43 to −0.43)^b^		0.42 (−0.59 to 1.43)	
	Grocery and pharmacy percent change from baseline	0.02 (−0.01 to 0.05)		0.02 (−0.01 to 0.05)	
	Residential percent change from baseline	0.05 (0.005 to 0.10)^b^		−0.07 (−0.12 to −0.02)^b^	
**19-24 years**					
	ARIMA (p,d,q)	ARIMA (1,0,0)		ARIMA (5,0,0)	
	School closure	−0.39 (−1.37 to 0.59)		1.44 (0.45 to 2.42)^b^	
	Grocery and pharmacy percent change from baseline	0.03 (0.005 to 0.06)^b^		0.01 (−0.01 to 0.04)	
	Residential percent change from baseline	0.01 (−0.04 to 0.05)		−0.04 (−0.09 to 0.005)	
**25-44 years**					
	ARIMA (p,d,q)	ARIMA (0,1,10)		ARIMA (0,0,1)	
	School closure	−0.21 (−1.47 to 1.05)		0.10 (−0.77 to 0.97)	
	Grocery and pharmacy percent change from baseline	0.05 (0.03 to 0.08)^b^		−0.02 (−0.04 to 0.01)	
	Residential percent change from baseline	0.01 (−0.02 to 0.05)		0.00 (−0.04 to 0.04)	
**45-64 years**					
	ARIMA (p,d,q)	ARIMA (6,1,2)		ARIMA (5,0,0)	
	School closure	−0.01 (−1.13 to 1.11)		0.26 (−0.67 to 1.18)	
	Grocery and pharmacy percent change from baseline	0.03 (0.01 to 0.06)^b^		0.02 (−0.01 to 0.04)	
	Residential percent change from baseline	0.07 (0.03 to 0.12)^b^		0.02 (−0.03 to 0.06)	
≥**65 years**					
	ARIMA (p,d,q)	ARIMA (7,0,0)		ARIMA (0,0,0)	
	School closure	−1.29 (−2.47 to −0.11)^b^		−0.21 (−1.24 to 0.83)	
	Grocery and pharmacy percent change from baseline	0.01 (−0.01 to 0.03)		0.00 (−0.03 to 0.03)	
	Residential percent change from baseline	0.12 (0.09 to 0.14)^b^		0.02 (−0.03 to 0.07)	

^a^ARIMA: autoregressive integrated moving average.

^b^*P*<.05.

**Table 5:** Effect of school closure on the standardized incidence of COVID-19–related syndromes in clinics and community hospitals from January 1, 2022, to June 6, 2022.

Age and variable			Clinic (estimation), coefficient (95% CI)		Hospital (estimation), coefficient (95% CI)
**Overall**					
	ARIMA^a^ (p,d,q)	ARIMA (8,0,0)		ARIMA (2,0,2)	
	School closure	−0.40 (−0.90 to 0.10)		−0.29 (−0.60 to 0.03)	
	Grocery and pharmacy percent change from baseline	0.03 (0.01 to 0.05)^b^		0.04 (0.02 to 0.06)^b^	
	Residential percent change from baseline	0.09 (0.04 to 0.14)^b^		0.14 (0.09 to 0.18)^b^	
**0-6 years**					
	ARIMA (p,d,q)	ARIMA (0,0,3)		ARIMA (5,0,0)	
	School closure	−0.23 (−0.63 to 0.18)		−0.09 (−0.18 to −0.01)^b^	
	Grocery and pharmacy percent change from baseline	0.01 (−0.01 to 0.03)		−0.01 (−0.01 to 0.000003)	
	Residential percent change from baseline	0.03 (−0.001 to 0.07)		0.03 (0.02 to 0.04)^b^	
**7-12 years**					
	ARIMA (p,d,q)	ARIMA (5,0,2)		ARIMA (6,0,2)	
	School closure	−0.05 (−0.54 to 0.45)		−0.25 (−0.50 to −0.01)^b^	
	Grocery and pharmacy percent change from baseline	0.00 (−0.02 to 0.02)		0.00 (−0.02 to 0.01)	
	Residential percent change from baseline	0.04 (−0.002 to 0.08)		0.07 (0.03 to 0.10)^b^	
**13-18 years**					
	ARIMA (p,d,q)	ARIMA (0,1,9)		ARIMA (10,0,0)	
	School closure	−0.35 (−0.74 to 0.04)		−0.05 (−0.32 to 0.23)	
	Grocery and pharmacy percent change from baseline	0.01 (−0.01 to 0.03)		0.01 (−0.01 to 0.02)	
	Residential percent change from baseline	−0.01 (−0.06 to 0.04)		0.02 (−0.02 to 0.05)	
**19-24 years**					
	ARIMA (p,d,q)	ARIMA (6,0,2)		ARIMA (7,0,0)	
	School closure	−0.31 (−0.80 to 0.17)		−0.38 (−0.74 to −0.03)^b^	
	Grocery and pharmacy percent change from baseline	−0.01 (−0.03 to 0.01)		−0.01 (−0.03 to 0.02)	
	Residential percent change from baseline	0.06 (0.02 to 0.10)^b^		0.09 (0.03 to 0.15)^b^	
**25-44 years**					
	ARIMA (p,d,q)	ARIMA (3,0,5)		ARIMA (7,0,0)	
	School closure	−0.23 (−0.68 to 0.22)		0.06 (−0.28 to 0.41)	
	Grocery and pharmacy percent change from baseline	0.02 (0.001 to 0.04)^b^		−0.01 (−0.03 to 0.01)	
	Residential percent change from baseline	0.07 (0.03 to 0.11)^b^		0.02 (−0.03 to 0.06)	
**45-64 years**					
	ARIMA (p,d,q)	ARIMA (6,1,0)		ARIMA (0,0,1)	
	School closure	−0.46 (−0.77 to −0.15)^b^		−0.22 (−0.48 to 0.05)	
	Grocery and pharmacy percent change from baseline	0.04 (0.02 to 0.05)^b^		0.04 (0.02 to 0.06)^b^	
	Residential percent change from baseline	0.10 (0.06 to 0.14)^b^		0.10 (0.06 to 0.13)^b^	
≥**65 years**					
	ARIMA (p,d,q)	ARIMA (7,0,0)		ARIMA (0,0,4)	
	School closure	−0.42 (−0.73 to −0.12)^b^		0.02 (−0.33 to 0.36)	
	Grocery and pharmacy percent change from baseline	0.04 (0.02 to 0.05)^b^		0.04 (0.01 to 0.06)^b^	
	Residential percent change from baseline	0.10 (0.06 to 0.14)^b^		0.08 (0.04 to 0.12)^b^	

^a^ARIMA: autoregressive integrated moving average.

^b^*P*<.05.

**Table 6:** Effect of school closure on the standardized incidence of COVID-19–related syndromes in clinics and community hospitals from January 1, 2022, to June 20, 2022.

Age and variable			Clinic (estimation), coefficient (95% CI)		Hospital (estimation), coefficient (95% CI)
**Overall**					
	ARIMA^a^ (p,d,q)	ARIMA (8,0,0)		ARIMA (0,0,8)	
	School closure	−0.29 (−0.77 to 0.19)		−0.19 (−0.48 to 0.10)	
	Grocery and pharmacy percent change from baseline	0.03 (0.02 to 0.05)^b^		0.04 (0.02 to 0.06)^b^	
	Residential percent change from baseline	0.09 (0.04 to 0.14)^b^		0.13 (0.09 to 0.17)^b^	
**0-6 years**					
	ARIMA (p,d,q)	ARIMA (7,0,0)		ARIMA (10,0,0)	
	School closure	−0.08 (−0.51 to 0.35)		−0.05 (−0.20 to 0.09)	
	Grocery and pharmacy percent change from baseline	0.00 (−0.01 to 0.02)		−0.01 (−0.02 to 0.00)	
	Residential percent change from baseline	0.05 (0.01 to 0.10)^b^		0.02 (0.01 to 0.04)^b^	
**7-12 years**					
	ARIMA (p,d,q)	ARIMA (7,0,0)		ARIMA (8,0,2)	
	School closure	0.05 (−0.39 to 0.48)		−0.29 (−0.61 to 0.04)	
	Grocery and pharmacy percent change from baseline	−0.01 (−0.02 to 0.01)		0.003 (−0.01 to 0.02)	
	Residential percent change from baseline	0.04 (0.001 to 0.08)^b^		0.02 (−0.01 to 0.06)	
**13-18 years**					
	ARIMA (p,d,q)	ARIMA (2,0,1)		ARIMA (10,0,0)	
	School closure	−0.26 (−0.70 to 0.19)		−0.02 (−0.31 to 0.26)	
	Grocery and pharmacy percent change from baseline	0.01 (−0.01 to 0.03)		0.01 (−0.01 to 0.02)	
	Residential percent change from baseline	0.00 (−0.04 to 0.04)		0.01 (−0.02 to 0.05)	
**19-24 years**					
	ARIMA (p,d,q)	ARIMA (3,0,3)		ARIMA (7,0,0)	
	School closure	−0.31 (−0.74 to 0.13)		−0.30 (−0.68 to 0.08)	
	Grocery and pharmacy percent change from baseline	−0.01 (−0.03 to 0.01)		−0.01 (−0.03 to 0.01)	
	Residential percent change from baseline	0.06 (0.03 to 0.10)^b^		0.03 (−0.01 to 0.08)	
**25-44 years**					
	ARIMA (p,d,q)	ARIMA (5,0,3)		ARIMA (7,0,0)	
	School closure	−0.17 (−0.61 to 0.28)		0.10 (−0.23 to 0.44)	
	Grocery and pharmacy percent change from baseline	0.02 (0.001 to 0.04)^b^		−0.01 (−0.03 to 0.01)	
	Residential percent change from baseline	0.06 (0.02 to 0.10)^b^		0.01 (−0.03 to 0.05)	
**45-64 years**					
	ARIMA (p,d,q)	ARIMA (6,1,0)		ARIMA (1,0,1)	
	School closure	−0.42 (−0.73 to −0.11)^b^		−0.14 (−0.38 to 0.11)	
	Grocery and pharmacy percent change from baseline	0.04 (0.02 to 0.05)^b^		0.04 (0.02 to 0.06)^b^	
	Residential percent change from baseline	0.10 (0.06 to 0.14)^b^		0.08 (0.05 to 0.11)^b^	
≥**65 years**					
	ARIMA (p,d,q)	ARIMA (7,0,0)		ARIMA (0,0,4)	
	School closure	−0.39 (−0.68 to −0.09)^b^		0.01 (−0.32 to 0.35)	
	Grocery and pharmacy percent change from baseline	0.04 (0.02 to 0.05)^b^		0.04 (0.01 to 0.06)^b^	
	Residential percent change from baseline	0.11 (0.07 to 0.15)^b^		0.08 (0.04 to 0.12)^b^	

^a^ARIMA: autoregressive integrated moving average.

^b^*P*<.05.

## Discussion

### Principal Findings

School closure was only significantly negatively associated with the overall standardized incidence of COVID-19–related syndromes in 2021 for 2 weeks after the intervention in community hospitals. However, within different age groups, the effects of school closure were not consistent. Among people aged 13-18 years and ≥65 years for 2 and 4 weeks after the intervention, school closure had a significantly negative association with the standardized incidence of COVID-19–related syndromes in 2021 in clinics. There were some suspected reasons for the effects of school closure. First, the scale of the epidemic was small in 2021, and the proportion of COVID-19–confirmed cases in children was low. School closure, including kindergarten closure, can prevent infection among students and children aged 2-6 years [26]. Second, both the COVID-19 alert level in 2021 and soft lockdown reduced population movements and COVID-19–confirmed cases especially among people aged ≥65 years. Social distancing actions contribute to the reduction of the number of new COVID-19 cases [27]. In Taiwan, some children live with not only their parents but also their grandparents. According to a 2017 report of the Senior Citizen Condition Survey, adults aged 55-64 years who take care of grandchildren account for 16.05% of the same age group, while daily care for grandchildren accounts for 10.79%. Adults aged 65 years or older who care for grandchildren account for 11.80% of the same age group [28]. School closure, which keeps students at home, has been linked to decreased COVID-19 incidence owing to this family structure. In community hospitals, school closure had a significantly positive association with the standardized incidence among people aged 19-24 years. We suspected that distance learning let college students have more freedom to arrange their time and have more opportunities for social contact. However, the relation between the contact rate and infection rate has to be explored. In a United States study, adults aged 20-34 years and 35-49 years had sustained SARS-CoV-2 transmission with reproduction numbers consistently above 1 as of October 29, 2020. The high transmission rates among adults aged 20-49 years are linked to both rebounding mobility over the summer and elevated transmission risk per venue visit [29].

In community hospitals in 2022, 2 weeks after the intervention among people aged 0-6, 7-12, and 19-24 years, the school closure had a significantly negative association with the standardized incidence of COVID-19–related syndromes. Because COVID-19 diagnoses were only given from hospitals or community screening stations before May 2022 and children died from COVID-19, the proportion of children visiting hospitals was high. Therefore, it is easier to observe the effects of school closure.

In 2022, school closure did not have an obvious effect on students after 4 weeks of the intervention. However, in clinics for people aged >45 years in 2022, school closure was significantly negatively associated with the standardized incidence of COVID-19–related syndromes. First, in the commuting zone of Taipei and New Taipei City, school closure was mandated for just 2 weeks, and the announcement was made by the Taipei government. Residents or students could still commute across cities and possibly become infected from the workplace or from crammed schools. Second, there was a general outbreak of epidemics, with the number of confirmed cases of COVID-19 rising after May 2022, which was tallied from Taiwan CDC [30]. Third, the alert level of COVID-19 was reduced from Level 3 to Level 2, and NPIs were less strict. Although school closure had no significant effect on students, it still had a protective effect on family members. A study in Sweden reported that exposure to open rather than closed schools resulted in a small increase in PCR-confirmed infections among parents (odds ratio 1.17, 95% CI 1.03-1.32) [31]. During school closure, students stay home, which reduces the risk of contact with schoolmates and indirectly reduces the risk of infection of family members, especially those who stay longer at home. School closure affects family routines, modified work schedules, remote work, and alternative childcare [32].

In terms of the hierarchy of medical facilities, children and young adults are more likely to visit clinics, while older people with comorbidities are more likely to visit community hospitals [33]. In addition, community hospitals are more likely to have cross-district patients. In Sentinel Plus, the proportion of patients diagnosed with COVID-19–related syndromes from community hospitals was low.

For people’s movement changes in 2021 and 2022 in different age groups, a longer grocery and pharmacy time was associated with a higher standardized incidence of COVID-19–related syndromes. This may be because early symptoms of infection are not obvious, and people may go to pharmacies to seek medication [34].

For people’s movement changes in 2022 in different age groups, a longer residence time was associated with a higher standardized incidence of COVID-19–related syndromes. First, the high rate of community infection and confirmed cases in 2022 could have easily caused household infections. This family structure increases the probability of household secondary transmission, and school closure, which keeps students at home, has been linked to increased COVID-19 incidence. A meta-analysis estimated the household secondary attack rate to be 16.6% (95% CI 14.0-19.3), which is higher than the secondary attack rates for SARS-CoV and MERS-CoV. The rates of household secondary attacks were higher for contacts with adults than for contacts with children [35]. Second, the alert level of COVID-19 was reduced from Level 3 to Level 2, relaxing some of the NPI policies.

During school closure in 2021, vaccinations were not administered. According to Taiwan CDC statistics [36], the vaccination rate for the first dose was 16.9% and that for the second dose was 0% among children aged 6-11 years. On May 23, 2022, the vaccination rate for the second dose was 82.1% among teenagers aged 12-17 years in Taipei City.

Since Taiwan did not implement a strict lockdown during the COVID-19 pandemic, Taiwanese citizens may have different fears, anxieties, and concerns compared to the citizens of other countries that implemented strict lockdown policies, leading to different attitudes and behaviors regarding infection risks [37]. Additionally, social media, as a platform for rapidly spreading information during disease outbreaks, may consequently influence public perceptions and emotions, potentially triggering behavior changes in response to the COVID-19 pandemic [38]. Under Taiwan’s soft lockdown policy, social and family compliance with epidemic prevention policies has played a great role in controlling the local epidemic. Overall, school closure was effective in suppressing COVID-19 among students due to reduced contact. A recent Taiwanese study constructed parameters of interpersonal contact patterns from diary-approach surveys. The results showed that the most active age group was schoolchildren (aged 5-14 years), with an average of 16-18 contacts [39]. Reducing schoolchildren’s contact rate can effectively reduce disease transmission. In addition, school closure has a spillover effect on elderly people who stay home. A systematic review of school closure studies showed substantial heterogeneity, with half of the studies at a lower risk of bias reporting a reduction in community transmission of up to 60% [40].

It is difficult to determine the specific impact of school closure on COVID-19 incidence when multiple nonpharmacological interventions are implemented simultaneously. Analysis of the 2022 time interval (the 10 months in which alert Level 2 lasted before the school closure policy was implemented) helps highlight the impact of school closure in Taipei City. Our outpatient syndromic surveillance system can divide the entire population into 7 age groups, allowing the differential impacts of school closure on different age groups to be explored. Specifically, because of the Chinese family structure with a higher proportion of grandparents, parents, and grandchildren living together in the same house, elderly people benefit from the protective effects of school closure.

### Comparison With Prior Work

Although some studies have explored the effect of school closure on COVID-19 infection, we focused on the effect in 7 different age groups with different severities of the epidemic. Furthermore, we used community-based syndromic surveillance to monitor immediate changes in COVID-19–related syndromes. Moreover, the movement of people to grocery stores, pharmacies, and residences was also considered.

Staguhn et al [26] noted that school closure significantly impacted COVID-19 infection rates in the United States. However, not all states were included in this study. Only states with state-wide stay-at-home orders, stay-at-home order dates preceding school closure dates, less than 3 days between the date of 10 cases and the school closure date, and less than 3 days between the school closure date and the stay-at-home order were included in the study. Stay-at-home orders that coincided with school closure may have influenced the effects observed in the data.

Another study analyzed nasopharyngeal swab-confirmed SARS-CoV-2 cases in Milan from February 1 to April 5, 2021. Total school closure in Lombardy was imposed between March 5 and April 5, 2021. The school closure intervention produced a daily reduction in the risk of contagion by 4% in those aged 3-11 years and 12-19 years, and by 3% in those aged ≥20 years. The epidemic curve decreased first in school-aged children after the intervention and subsequently in the adult population [41].

### Limitations

There are some limitations in this study. First, we used the standardized incidence of COVID-19–related syndromes as an indicator and not confirmed cases of COVID-19. Before the end of May 2022, COVID-19 cases were only diagnosed by COVID-19 PCR tests from community screening stations and emergency departments of hospitals. The COVID-19–related syndromic group was first established in November 2020. Although COVID-19 presenting symptoms changed with the evolution of coronavirus variants, we discussed with infectious disease physicians and family doctors a few months after the COVID-19 pandemic in 2020, based on their clinical observations and studies, to define susceptible patients at that time. Some symptoms may vary with variants; however, it remains difficult to make a differential diagnosis. For our purposes, if we could find one symptom of a COVID-19–related syndrome, we accounted for it in our model. In the future, a dynamic definition of syndrome groups is needed to update the clinical manifestations of the disease. Second, during the periods of school closure in 2021 and 2022, positive rates for influenza or respiratory viral infections were nearly zero, and positive cases for respiratory viral infections in 2021 throughout the year were very low (828 cases collected in contracted laboratories for viral infection symptoms) [42]. COVID-19–related syndromes are important diagnoses of potential infections in the community. Third, the data set of people’s movement included national data, but Sentinel Plus was only for Taipei. Not everyone, especially children, had mobile devices. The accuracy of the children’s movement changes needs to be further explored. Fourth, potential confounders, such as family structures, vaccination status, citizens’ perceptions and emotions, and the influence of media, which can influence social networks, infection rates, immunity levels, and behavior changes, were not considered owing to the limitations of aggregated surveillance data. However, our intervention period of school closure was short, and confounding factors may not have changed much during the intervention period.

### Conclusions

Nationwide school closure, encouragement to work from home, and COVID-19 alert Level 3 were implemented in 2021. Although vaccine coverage was zero in the first observation period in 2021 among the student and preschool populations, the effect of school closure was observed among them. In 2022, the COVID-19 alert was downgraded to Level 2, there was no consistent implementation of school closure policies in each county or city, and people could not work from home unless they requested unpaid family care leave. However, an effect of school closure was also found in the student population after 2 weeks of the intervention. We concluded that the early stage of the COVID-19 outbreak and the short intervention period (2 weeks) effectively prevented and postponed student infection. However, if the epidemic is widespread, school closure alone is not effective in reducing transmission.

Dynamic and real-time surveillance can offer practical policy recommendations to local health departments, enabling them to proactively address the risk of disease transmission within communities. Various policy tools are currently in use to control epidemics, including health education via social media and mass media, as well as measures like class suspension and school closure. By using a community-based surveillance system, aberrant signals can be accurately tailored to specific age groups and geographical locations, allowing for early detection and affording additional time for the implementation of further interventions. This proposed approach is not limited to COVID-19 and can be extended to manage other infectious diseases such as influenza and enterovirus.

## Abbreviations

AIC: Akaike information criterion
ARIMA: autoregressive integrated moving average
ARIMAITS-DL: autoregressive integrated moving average interrupted time series- distributed lag
CDC: Centers for Disease Control
ICD-10: International Classification of Diseases, 10th Revision
NPI: nonpharmaceutical intervention
PCR: polymerase chain reaction

## Data Availability

The data used are provided on the open repository Figshare [43].
